# Determinants of anemia among pregnant women attending a tertiary hospital, Mogadishu, Somalia: unmatched case-control study

**DOI:** 10.1590/1806-9282.20231376

**Published:** 2024-05-17

**Authors:** Marian Muse Osman, Esra Keles, Guven Bektemur, Hasan Huseyin Eker, Şeyma Karaketir, Ozgur Ozer

**Affiliations:** 1 Somalia National Institute of Health, Research Department – Mogadishu, Somalia.; 2 University of Health Sciences Turkey, Hamidiye Faculty of Medicine, Department of Public Health – İstanbul, Turkey.; 3 University of Health Sciences Turkey, Kartal Dr. Lütfi Kırdar City Hospital, Department of Gynaecologic Oncology – İstanbul, Turkey.; 4 University of Health Sciences Turkey, Mogadishu Somalia-Turkey Recep Tayyip Erdoğan Training and Research Hospital, Department of Public Health – Mogadishu, Somalia.; 5 Istanbul University, Istanbul School of Medicine, Department of Public Health, Occupational Health Training Programme – İstanbul, Turkey.; 6 University of Health Sciences Turkey, Mogadishu Somalia-Turkey Recep Tayyip Erdoğan Training and Research Hospital, Department of Obstetrics and Gynecology – Mogadishu, Somalia.

**Keywords:** Risk factors, Anemia, Pregnant women, Diet, Antenatal care, Somalia

## Abstract

**OBJECTIVE:**

The objective of this study was to identify the factors associated with anemia among pregnant women attending a tertiary referral hospital in Mogadishu, Somalia.

**METHODS:**

An unmatched case-control study was conducted on pregnant women who visited the antenatal clinics of a tertiary referral hospital between March and July 2021. The study recruited pregnant women who had a hemoglobin level of <11 g/dL into the anemic group, while those with hemoglobin levels ≥11 g/dL were included in the non-anemic group. Demographics, clinical, obstetrics, nutrition-related, hygiene- and sanitation-related, and parasitic infection-related data were collected.

**RESULTS:**

A total of 449 pregnant women (399 anemic and 50 non-anemic) participated in the study. A total of 224 (56.7%) in the anemic group and 31 (62.0%) in the non-anemic group did not consume any dark green, leafy vegetables such as spinach, bukurey, cagaar, and koomboow (p=0.040). Notably, 255 (63.9%) in the anemic group and 21 (42.0%) in the non-anemic group had a middle-upper arm circumference <23 cm. More than half of anemic [335 (84%)] and non-anemic [46 (92.0%)] were classified under low dietary diversity score. Majority of the study participants, 288 (72.4%) of the anemic and 39 (78%) of the non-anemic groups, used pit toilets in dwellings, and 70.2% (134/191) of the anemic and 64.4% (246/382) of the non-anemic groups disposed of solid waste in open fields.

**CONCLUSION:**

This study demonstrated that women who consumed green vegetables such as spinach, bukurey, cagaar, and koomboow in their diet had middle-upper arm circumference less than 23 cm, and those with low dietary diversity significantly developed anemia during pregnancy.

## INTRODUCTION

Anemia is a public health concern that affects nearly one-third of the global population, with higher rates among young, impoverished, and pregnant women^1^. It is a significant cause of maternal and fetal morbidity and mortality, especially in low-resourced countries^2^.

The primary cause of anemia is iron deficiency, although other deficiencies, diseases, and inherited blood disorders may also contribute. Risk factors for anemia can be individual, household, community, district, regional, or national^3^. Globally, anemia results in over 115,000 maternal and 591,000 perinatal deaths annually, accounting for 20% of maternal deaths worldwide^4^. Developing countries have higher rates of anemia among pregnant women, with Africa and Southeast Asia having the highest rates^5^. In Somalia, maternal anemia is highly prevalent, with a rate of 49.9%, especially in rural areas^6^.

The World Health Organization (WHO) aims to reduce anemia in reproductive-aged women by 50% by 2025^7^. Maternal nutrition is a key priority in the Health Sector Transformation Plan (HSTP), and the prevalence of anemia in reproductive-aged women is one of the outcome measures of HSTP targets^8^. However, there are currently no reported studies on anemia among pregnant women in Somalia. Therefore, in resource-limited settings like Somalia, it is critical to obtain recent and accurate data on anemia severity and associated factors in order to meet goals and prioritize those most at risk.

This study aimed to identify the factors that contribute to anemia in pregnant women who sought care at a tertiary referral hospital in Somalia, where there is less evidence available on this issue. By identifying the risk factors associated with anemia, healthcare providers can prioritize interventions that will benefit the most vulnerable women and reduce the burden of anemia-related morbidity and mortality.

## METHODS

An institution-based unmatched case-control study was carried out on pregnant women who visited the antenatal clinics of Mogadishu Somalia Turkish Training and Research Hospital between March and July 2021. The research received approval from the Ethical Committee of the hospital and adhered to the tenets of the Declaration of Helsinki (Approval number: 17.02.2021- MSTH/5462). Pregnant women were provided information on the voluntary nature of the study, and written consent was obtained.

Pregnant women who suffered from acute or chronic disease, were receiving anemia treatment, were receiving deworming medication within the past 4 weeks, and those who could not provide information were excluded.

Anemia in pregnancy was determined following the guidelines provided by the WHO^9^. Pregnant women who had a hemoglobin level of <11 g/dL were included in the anemia group, while those with hemoglobin levels ≥11 g/dL were included in the non-anemic group. Hemoglobin levels were measured using a Sysmex XN 9000 (Roche Diagnostics Indianapolis, IN) analyzer. The blood samples were collected, processed, and analyzed by trained and experienced laboratory technicians and healthcare professionals with research experience. Data were collected using a questionnaire based on the existing literature and adapted to this study. Demographics, clinical, obstetrics, nutritional, hygiene- and sanitation-related, and parasitic infection-related data were gathered through face-to-face interviews with trained research assistants. To ensure the quality of data, data collectors were supervised by the principal investigator. All filled questionnaires were checked on a daily basis for completeness and consistency.

The study used the dietary diversity questionnaire (DDQ) to evaluate the usual dietary intakes of pregnant women in the last 6 months. Minimum Dietary Diversity for Women (MDD-W) was assessed by 24-h dietary recall. Dietary diversity was classified into low (<3), medium^4-6^, and high (<7)^10^. The nutritional status of pregnant women was assessed by measuring mid-upper arm circumference (MUAC) with a tape measure by the trained research assistant. A MUAC <23 cm was indicative of malnutrition^11^.

### Statistical analysis

Data were analyzed through Statistical Package for Social Sciences (SPSS) v.20.0. Descriptive statistics were utilized to calculate frequencies and percentages, with p≤0.05 considered significant. Statistical analysis was done using the chi-square test, Fisher’s exact test, or Mann-Whitney test, as appropriate.

## RESULTS

### Socioeconomic characteristics

The study involved 449 pregnant women, out of which 399 had anemia and 50 served as the non-anemic group. The mean age in the anemic and non-anemic groups were 25 (±6.0) and 25.0 (±5) years, respectively. The vast majority of participants resided in urban areas, with 93.9% in the anemic group and 98% in the non-anemic group. Notably, 3% of the anemic group was from rural areas. Regarding occupational status, most participants were housewives, with 317 (79.4%) in the anemic group and 38 (76%) in the non-anemic group. Family size was greater than nine for over half of the participants, with 50.4% in the anemic group and 51.0% in the non-anemic group. A total of 216 (55.2%) participants in the anemic group and 35 (70.0%) non-anemic group had a monthly income ranging between 300 and 500 USD (Table 1).

**Table 1:** Baseline characteristics (n=449).

Variables		Anemic group (n=399)		Non-anemic group (n=50)		p-value
		n	%	n	%	
Age (years)	Mean±SD/median (range)	25±6/25 (25)		25±5/25 (24)		0.959
Age (years)	15–19	66	16.6	5	10.0	0.395
	20–24	111	27.9	20	40.0	
	25–29	123	30.9	13	26.0	
	30–34	66	16.6	9	18.0	
	35–40	32	8.0	3	6.0	
Marital status	Married	368	93.2	45	93.8	0.201
	Widowed	5	1.3	2	4.2	
	Divorced	22	5.6	1	2.1	
Residence	IDP	11	2.8	1	2.0	0.599
	Rural	13	3.3	0	.0	
	Urban	372	93.9	49	98.0	
Family size	<9	192	49.6	24	49.0	0.933
	=9	195	50.4	25	51.0	
Occupation of respondent	Daily laborer	30	7.5	3	6.0	0.780
	Government employee	17	4.3	3	6.0	
	Housewife	317	79.4	38	76.0	
	Private employee	11	2.8	2	4.0	
	Student	24	6.0	4	8.0	
Occupation of spouse	Daily laborer	188	47.2	29	59.2	0.451
	Government employee	85	21.4	12	24.5	
	Merchant	48	12.1	3	6.1	
	Private employee	41	10.3	4	8.2	
	Farmer/pastoralist	7	1.8	0	0	
	Unemployment	29	7.3	1	2.0	
Income	Poor (<100 USD)	15	3.8	0	0	0.084
	Middle (100–300 USD)	160	40.9	15	30.0	
	High (300–500 USD)	216	55.2	35	70.0	
Educational status	No formal school	137	34.5	17	34.0	1.000
	Illiterate	8	2.0	1	2.0	
	Primary school education (Grades 1–9)	130	32.7	16	32.0	
	Secondary school education (Grades 10–12)	86	21.7	11	22.0	
	Tertiary (college/university)	36	9.1	5	10.0	

### Anthropometric characteristics

The majority of the pregnant women involved in the study were malnourished, with 255 (63.9%) in the anemic group and 21 (42.0%) in the non-anemic group having a MUAC of less than 23 cm (Table 2).

**Table 2:** Health conditions and anthropometric characteristics of pregnant women attending antenatal care in Mogadishu Somalia Training and Research Hospital, 2021 (n=449).

Variables		Anemic group (n=399)		Non-anemic group (n=50)		p-value
		n	%	n	%	
Interval between pregnancies	<2 years	320	97.9	41	100.0	1.000
	≥2 years	7	2.1	0	0	
Antenatal follow-up	<4	382	95.7	49	98.0	
	≥4	17	4.3	1	2.0	
Tetanus vaccination	No	138	34.8	19	38.0	0.867
	Incomplete	252	63.6	30	60.0	
	Complete	6	1.5	1	2.0	
Iron supplementation	No	134	33.6	16	32.0	0.823
	Yes	265	66.4	34	68.0	
Folic acid supplementation	No	173	43.5	20	40.0	0.641
	Yes	225	56.5	30	60.0	
Treatment for intestinal worms	No	374	94.2	47	95.9	1.000
	Yes	23	5.8	2	4.1	
Treatment for malaria infection	No	382	96.2	48	96.0	1.000
	Yes	15	3.8	2	4.0	
Smoking	No	395	100.0	50	100.0	na
	Yes	0	.0	0	.0	
Menstrual period (days)	<8	366	92.7	45	90.0	0.568
	≥8	29	7.3	5	10.0	
MUAC	<23 cm	255	63.9	21	42.0	0.003*
	≥23 cm	144	36.1	29	58.0	
Dietary diversity score	Low	335	84.6	46	92.0	0.376
	Medium	45	11.4	3	6.0	
	High	16	4.0	1	2.0	

MUAC: mid-upper arm circumference; CS: cesarean section; NSD: normal spontaneous vaginal delivery;

*p<0.05.

### Maternal dietary factors

More than half of the anemic [335 (84%)] and non-anemic groups [46(92.0%)] had a low dietary diversity score, whereas 45 (11.4%) and 16 (4.0%) of the anemic group and 3 (6%) and 1 (2.0%) of the non-anemic group had medium and high dietary diversity scores, respectively (Table 2).

### Maternal obstetric factors

The pregnancy interval for most study participants was less than 2 years, with 320 (97.9%) of the anemic group and 41 (100%) of the non-anemic group. The anemic group with menstrual bleeding more than 8 days per cycle was 29 (7.3%), while it was 5 (10%) in the non-anemic group. Those who attended antenatal visits less than four times during their pregnancies were 17 (4.3%) of the anemic women and 1 (2.0%) of the non-anemic group (Table 2).

### Hygiene- and sanitation-related factors

A total of 288 (72.4%) of the anemic group and 39 (78%) of the non-anemic group used pit toilets in dwellings. Notably, 70% (134/191) of the anemic group and 64.4% (246/382) of the non-anemic group stated that they disposed of solid waste in open fields. A total of 158 (98.8%) of the anemic group and 23 (100%) of the non-anemic group had access to protected piped water as a source of drinking water (Table 3).

**Table 3:** Hygiene- and sanitation-related factors of anemia in pregnant women attended antenatal care in Mogadishu Somalia Training and Research Hospital, 2021 (n=449).

Variables		Anemic group		Non-anemic group	
		n	%	n	%
Source of drinking water	Protected piped water into dwelling	159	98.8	23	100.0
	Unprotected piped water into dwelling	2	1.2	0	0
Toilet facility	Composed latrines	2	0.5	0	0
	Flush toilet in dwelling	105	26.4	11	22.0
	No facility/bush/field	3	0.8	0	0
	Pit toilet in dwelling	288	72.4	39	78.0

### Nutrient intake

Examination of the nutrition intake showed that 224 (56.7%) of the anemic group and 31 (62.0%) non-anemic group did not consume any dark green, leafy vegetables such as spinach, bukurey, cagaar, and koomboow (p=0.040). A total of 181 (45.6%) anemic and 25 (50.0%) non-anemic participants did not eat fresh or dried fish or shellfish, and 208 (52.3%) of the anemic and 20 (40%) of the non-anemic groups did not consume any meat, such as beef, lamb, goat, chicken, or duck at all. Totally, 159 (39.8%) of those with anemia and 20 (40.0%) of the non-anemic group did not consume injera, muufo, soor, bread, rice, noodles, spaghetti, porridge, or other foods made from grains such as oats, maize, and barley. Notably, 203 (50.9%) of the anemic group and 29 (58.0%) of the non-anemic group never consumed any foods made from beans, peas, lentils, or nuts. A total of 209 (52.6%) of the anemic group and 18 (36.0%) of the non-anemic group did not consume yogurt, cheese, or other food made from milk.

## DISCUSSION

Anemia is a major global public health issue, particularly during pregnancy in developing nations such as Somalia. This study aimed to identify the factors contributing to anemia among pregnant women who were admitted to the obstetrics unit in Mogadishu, Somalia.

The study found that anemia was more prevalent among pregnant women who consumed dark green, leafy vegetables such as spinach, bukurey, cagaar, and koomboow compared with those who did not consume green leafy vegetables. This finding is in contrast to previous studies in Dessie, northern and eastern Ethiopia, which identified low intake of green vegetables as a contributing factor to anemia^12-14^. Food taboos during pregnancy, socioeconomic factors, climatic conditions, and inadequate dietary diversity may contribute to anemia in Somalia^3,15^.

This study found that a MUAC of less than 23 cm was significantly associated with anemia during pregnancy. MUAC is a common metric for assessing nutritional status, and a circumference of 23 cm serves as a threshold for determining the level of nourishment for women, with circumferences below this value being indicative of undernourishment. The result of this study aligns with prior research conducted in various regions, including eastern and western Ethiopia, Kenya, Nepal, and India^12,16,17^. These studies further demonstrate that undernourished pregnant women are more likely to develop anemia. The nutritional disadvantage of pregnancy might manifest itself later in pregnancy, as mothers are unable to provide the nutritional needs of the growing fetus, which may lead to negative consequences for both the mother and the fetus.

Dietary diversity is used as an indicator of the nutritional quality of pregnant women. This study found that a majority of pregnant women had a low dietary diversity score, which could be attributed to restrictive dietary behavior in Somalia where women are expected to avoid some foods during pregnancy. This, in turn, may contribute to anemia^18^. It is advisable to diversify the diet during pregnancy to meet the high nutritional needs.

### Limitations

The study had some limitations. First, it is a facility-based study, so it is difficult to generalize the findings to the other settings of Somalia or to other countries. Second, the cross-sectional design makes it difficult to draw causal inferences. Third, 24-h recall data might be subject to some recall bias. Finally, there might be social desirability bias in self-reports of dietary intake and household income. Despite having these limitations, to the best of our knowledge, this is the first comprehensive research on anemia among pregnant women to be reported from Mogadishu, Somalia.

## CONCLUSION

This study demonstrated that consumption of dark green, leafy vegetables such as spinach, bukurey, cagaar, and koomboow, low dietary diversity, and a MUAC less than 23 cm were found to be significant determinants of anemia among pregnant women in Somalia. Identifying and addressing the determinants of anemia and effective interventions are crucial to combat anemia in pregnancy.

## References

[1] World Health Organization Anaemia.

[2] Siteti MC, Namaska DN, Ariya OP, Injete SD, Wanyonyi WA (2014). Anaemia in pregnancy: prevalence and possible risk factors in Kakamega County, Kenya. Sci J Public Health.

[3] Tirore LL, Mulugeta A, Belachew AB, Gebrehaweria M, Sahilemichael A, Erkalo D (2021). Factors associated with anaemia among women of reproductive age in Ethiopia: multilevel ordinal logistic regression analysis. Matern Child Nutr.

[4] Kejela G, Wakgari A, Tesfaye T, Turi E, Adugna M, Alemu N (2020). Prevalence of anemia and its associated factors among pregnant women attending antenatal care follow up at Wollega University referral hospital, Western Ethiopia. Contracept Reprod Med.

[5] Liyew AM, Tesema GA, Alamneh TS, Worku MG, Teshale AB, Alem AZ (2021). Prevalence and determinants of anemia among pregnant women in East Africa; a multi-level analysis of recent demographic and health surveys. PLoS One.

[6] The World Bank (2018). Prevalence of anemia in women of reproductive age, WHO, global health observatory data repository, world health statistics.

[7] World Health Organization (2014). Essential nutrition actions: improving maternal, newborn, infant and young child health and nutrition.

[8] Federal Ministry of Health (2015). Health sector transformation plan.

[9] World Health Organization (2011). Vitamin and mineral nutrition information system.

[10] FAO & FANTA (2014). New dietary diversity indicator for women.

[11] Ververs MT, Antierens A, Sackl A, Staderini N, Captier V (2013). Which anthropometric indicators identify a pregnant woman as acutely malnourished and predict adverse birth outcomes in the humanitarian context?. PLoS Curr.

[12] Osman MO, Nour TY, Bashir HM, Roble AK, Nur AM, Abdilahi AO (2020). Risk factors for anemia among pregnant women attending the antenatal care unit in selected Jigjiga public health facilities, Somali Region, East Ethiopia 2019: unmatched case-control study. J Multidiscip Healthc.

[13] Tadesse SE, Seid O, G/Mariam Y, Fekadu A, Wasihun Y, Endris K (2017). Determinants of anemia among pregnant mothers attending antenatal care in Dessie town health facilities, northern central Ethiopia, unmatched case -control study. PLoS One.

[14] Egbi G, Gbogbo S, Mensah GE, Glover-Amengor M, Steiner-Asiedu M (2018). Effect of green leafy vegetables powder on anaemia and vitamin-A status of Ghanaian school children. BMC Nutr.

[15] Ahmed RH, Yussuf AA, Ali AA, Iyow SN, Abdulahi M, Mohamed LM (2021). Anemia among pregnant women in internally displaced camps in Mogadishu, Somalia: a cross-sectional study on prevalence, severity and associated risk factors. BMC Pregnancy Childbirth.

[16] Tulu BD, Atomssa EM, Mengist HM (2019). Determinants of anemia among pregnant women attending antenatal care in Horo Guduru Wollega Zone, West Ethiopia: unmatched case-control study. PLoS One.

[17] Okube OT, Mirie W, Odhiambo E, Sabina W, Habtu M (2016). Prevalence and factors associated with anaemia among pregnant women attending antenatal clinic in the second and third trimesters at Pumwani maternity hospital, Kenya. J Obstet Gynecol.

[18] Mengie T, Dessie Y, Egata G, Muche T, Habtegiorgis SD, Getacher L (2022). Food taboos and associated factors among agro-pastoralist pregnant women: a community-based cross-sectional study in Eastern Ethiopia. Heliyon.

